# Wisconsin State Medical Society

**Published:** 1871-08

**Authors:** 


### WISCONSIN STATE MEDICAL SOCIETY.

The Wisconsin State Medical Society held its 25th Annual Session in the city of Milwaukee, on the 21st, 22d and 23d days of June—its President, Dr. H. P. Strong, of Beloit, presiding.

After being opened with prayer by the Rev. J. L. Dudley, it was addressed by Dr. S. Marks, Chairman of the Committee of Arrangements, who, in behalf of the Committee, welcomed the Society to the city.

The calling of the roll by the Secretary showed a large attendance of the members, and the session was in all respects a harmonious, pleasant and profitable one.

A number of valuable papers were read, the first of which was from Dr. Marks, Chairman of the Committee on Surgery, on the Treatment of Fractures, based on cases which had passed under his observation during the past year, following which there arose an interesting and animated discussion concerning the use of carbolic acid in wounds, the opinion being given by President Strong and several others, that, like all other new remedies, the tendency was to a too common and indiscriminate use of the acid, sometimes to the destruction of healthy granulations.

Dr. Mason and Dalton each referred to the fact that the acid had been found a nice test for albumen, and that the surface of wounds dressed with it were often found coated with an albuminous deposit.

Dr. Wight called attention to the fact that castor oil was always preferable to glycerine as a menstruum, for the acid glycerine having the property of drying the tissues.

Dr. Page read a paper on the Treatment of Fevers.

Dr. Wight read a long and valuable paper on Medical Education, advocating more thorough preliminary education, and a lengthened term of study, which should be not less than five years, the whole of which should be passed in the Medical College, no deviation from which should be permitted except under the most stringent rules, "in which an exhaustive examination is made by a joint committee from the faculty of the Medical School, and from the class in which admission is sought."

Under the influence of such a thorough system of education, he claims that Medical Sectarianism would be more likely to die out, and the confidence of the people being strengthened in the regular profession, quackery of all kinds would cease to flourish.

Dr. J. K. Bartlett read a valuable paper, on Vesico-Vaginal Fistula, giving an historical sketch of the methods which have been resorted to for the cure of that malady.

At the opening of the afternoon session of the 22d, the Annual Address, by President Strong, was delivered, on "Conservatism in Medicine," for which able, eloquent and interesting address he received a special vote of thanks from the Society.

Dr. Armstrong read a very valuable essay on "The Cause and Treatment of Puerperal Convulsions, and the Condition of the Puerperal Woman."

Dr. Russell, from Committee on New Remedies, made a report, touching on Chloral Hydrate, the Ordeal Bean of Calibar, Iodoform, Apiol, Carbolic Acid, and Nitrous Oxide.

Dr. E. W. Bartlett read a paper on "The Accommodation of the Eye."

Dr. D. C. Davies, Special Committee on Gynæcology, made an interesting report.

Dr. Johnson, read a paper on "The Unity of Disease," and Dr. Armstrong reported a case of Aneurism of the Internal Iliac Artery."

Amendments to the Constitution were proposed, lengthening the term of office of the Censors, and providing for the examination by them of candidates for the study of medicine, as to their educational qualifications, and that no student shall be received into the office of a member of this Society until such student shall present the Censor's certificate that he has the requisite preliminary education.

The proposed amendments lie over for one year.

In the evening session of June 22d, the first business being to fix a place for the next meeting, Fond du Lac was chosen, when the Society proceeded to elect officers for the ensuing year, with the following result:

*President*—Dr. John Favill, of Madison.

*1st Vice-Pres't*—Dr. M. Waterhouse, of Portage.

*2d Vice-Pres't*—Dr. J. K. Bartlett, of Milwaukee.

*Secretary and Treasurer*—Dr. J. T. Reeve, of Appleton.

*Ass't-Secretary*—Dr. Theron Nichols, of Milwaukee.

*Censors*—Drs. J. K. Bartlett, Harmon Van Dusen, and Darius Mason.

The following named gentlemen were then elected Delegates to the next meeting of the American Medical Association—to wit:

Drs. J. K. Bartlett, J. G. Meachem, J. L. Brenton, J. T. Reeve, H. Van Dusen, A. H. Van Norstraud, O. W. Wight, S. Marks, G. F. Witter, Ch. Linde, M. Waterhouse, H. O. Crane, H. Palmer, E. B. Wolcott, H. P. Strong, and D. M. Bond.

After electing Delegates to several of the State Societies, the further reading of Essays and Reports was in order, when Dr. Palmer reported a case of "Subcutaneous Nevus successfully treated by Injections of Persulphate of Iron," exhibiting photographs of the case.

Dr. Griffin read an eloquent eulogy on the late lamented Dr.

Henry M. Lilly, of Fond du Lac, one of our most valued members, presenting to the Society, in the name of the Fond du Lac Co. Medical Society, a photograph of the deceased.

The "Synopsis of a case of Diseased Bladder and Kidney, resulting from Enlarged Prostate," was furnished by Dr. Godfrey; after which the Society adjourned.

The last morning's Session was a busy one, a number of valuable papers being read and referred to the Publication Committee, of which only the titles can here be given:

Dr. Linde read the report of some cases in Obstetrical Practice.

Dr. Cory read an essay on "Hysterico-Neuralgia, or Spinal Irritation, with report of Cases."

Dr. Kempe read the history of a case in which a wooden pessary had been retained for over seventeen years, and was removed with great difficulty, and only by being cut in pieces.

Dr. Senn reported a case of "Excision of the Clavicle for Osteo-Sarcoma."

Dr. Moore reported a case in which the operation of paracentesis thoracis had been performed by him.

Dr. Armstrong reported a case of "Abscess of the Right Ovary."

Dr. Witter reported a case of "Recto-Vaginal Fistula in an Infant."

Dr. Chase reported "A Case of Ovariotomy."

Dr. Ferrin reported "A Case of Complete Inversion of the Bladder, and Protrusion of that Organ at the Uretha."

A valuable communication on the subject of Puerperal Convulsions was received from Dr. Wilber, a former member of the Society, which was referred to the Committee on Publication.

The following list of Special Committees, and of appointments under them, was then made:

*On Skin Diseases*—Dr. C. Linde, Committee.

*On Diseases of the Brain and Nervous System, including Insanity*—Drs. S. L. Fuller and M. Waterhouse, Committee.

*On Diseases of the Respiratory Organs*—Dr. T. Nichols, Committee.

*On Indigenous Medical Botany*—Dr. N. Senn, Committee.

Resolutions of thanks to the Committee of Arrangements, to the officers of the Society, and to the Press were adopted.

A Committee of ten was appointed on Climatology and Prevailing Diseases; Dr. J. K. Bartlett, of Milwaukee, Chairman.

The President elect was conducted to the chair, and announced the following as the Standing Committees for the ensuing year:

*Arrangements*—Drs. Griffin, Mayham, and Barnes.
*Surgery*—Drs. Mason, Meacher, and Senn.
*Practice*—Drs. Mauley, Fox, and Ferrin.
*Obstetrics*—Drs. Armstrong, Reynolds, and Butterfield.
*Pathology*—Drs. J. Johnson, J. G. Meacham, and Rice.
*New Remedies*—Drs. Brown, Jenkins, and Dodson.
*Medical Education*—Drs. J. E. Davies and Bunnell.
*Diseases of the Eye*—Dr. E. W. Bartlett.

Dr. Griffin was requested to prepare an obituary notice of the late Dr. M. K. Darling, of Fond du Lac, the first President of this Society; after which the Society adjourned, to meet in Fond du Lac on the third Wednesday in June, 1872.

Following the adjournment a number of members of the Society spent a pleasant hour in visiting the National Asylum for Disabled Volunteer Soldiers, which is situated a few miles out of the City, a special train having been kindly provided for the purpose. J. T. R.

